# The recurrent missense mutation p.(Arg367Trp) in YARS1 causes a distinct neurodevelopmental phenotype

**DOI:** 10.1007/s00109-021-02124-9

**Published:** 2021-09-18

**Authors:** Luisa Averdunk, Heinrich Sticht, Harald Surowy, Hermann-Josef Lüdecke, Margarete Koch-Hogrebe, Hessa S. Alsaif, Kimia Kahrizi, Hamad Alzaidan, Bashayer S. Alawam, Mohamed Tohary, Cornelia Kraus, Sabine Endele, Erin Wadman, Julie D. Kaplan, Stephanie Efthymiou, Hossein Najmabadi, André Reis, Fowzan S. Alkuraya, Dagmar Wieczorek

**Affiliations:** 1 Institute of Human Genetics, Medical Faculty and University Hospital Düsseldorf, Heinrich-Heine-University Düsseldorf, Universitätsstraße 1, 40225 Düsseldorf, Germany; 2 Department of General Pediatrics, Neonatology and Pediatric Cardiology, Medical Faculty and University Hospital Düsseldorf, Heinrich-Heine-University, Düsseldorf, Germany; 3 Division of Bioinformatics, Institute of Biochemistry, Friedrich-Alexander-Universität Erlangen-Nürnberg, Erlangen, Germany; 4 Children’s Hospital Datteln, University Witten Herdecke, Datteln, Germany; 5 Department of Genetics, King Faisal Specialist Hospital and Research Center, Riyadh, Saudi Arabia; 6 Genetics Research Center, University of Social Welfare and Rehabilitation Sciences, Tehran, Iran; 7 Institute of Human Genetics, University Hospital Erlangen, Friedrich-Alexander-Universität Erlangen-Nürnberg, Erlangen, Germany; 8 Division of Medical Genetics, Department of Pediatrics, Nemours Alfred I, DuPont Hospital for Children, Wilmington, Delaware, DE USA; 9 Department of Neuromuscular Diseases, UCL Queen Square Institute of Neurology and The National Hospital for Neurology and Neurosurgery, London, UK

**Keywords:** Functional protein domains, Phenotypic heterogeneity, Aminoacyl-tRNA synthetases (ARS1), Novel disease genes, Neurodevelopmental disorders, Multisystem diseases

## Abstract

**Abstract:**

Pathogenic variants in aminoacyl-tRNA synthetases (ARS1) cause a diverse spectrum of autosomal recessive disorders. Tyrosyl tRNA synthetase (TyrRS) is encoded by *YARS1 (*cytosolic*,* OMIM*603,623*)* and is responsible of coupling tyrosine to its specific tRNA. Next to the enzymatic domain, TyrRS has two additional functional domains (N-Terminal TyrRS^Mini^ and C-terminal EMAP-II-like domain) which confer cytokine-like functions. Mutations in *YARS1* have been associated with autosomal-dominant Charcot-Marie-Tooth (CMT) neuropathy type C and a heterogenous group of autosomal recessive, multisystem diseases. We identified 12 individuals from 6 families with the recurrent homozygous missense variant c.1099C > T;p.(Arg367Trp) (NM_003680.3) in *YARS1*. This variant causes a multisystem disorder with developmental delay, microcephaly, failure to thrive, short stature, muscular hypotonia, ataxia, brain anomalies, microcytic anemia, hepatomegaly, and hypothyroidism. *In silico* analyses show that the p.(Arg367Trp) does not affect the catalytic domain responsible of enzymatic coupling, but destabilizes the cytokine-like C-terminal domain. The phenotype associated with p.(Arg367Trp) is distinct from the other biallelic pathogenic variants that reside in different functional domains of TyrRS which all show some common, but also divergent clinical signs [(e.g., p.(Phe269Ser)—retinal anomalies, p.(Pro213Leu)/p.(Gly525Arg)—mild ID, p.(Pro167Thr)—high fatality)]. The diverse clinical spectrum of ARS1-associated disorders is related to mutations affecting the various non-canonical domains of ARS1, and impaired protein translation is likely not the exclusive disease-causing mechanism of *YARS1*- and ARS1-associated neurodevelopmental disorders.

**Key messages:**

- The missense variant p.(Arg367Trp) in YARS1 causes a distinct multisystem disorder.
- p.(Arg367Trp) affects a non-canonical domain with cytokine-like functions.
- Phenotypic heterogeneity associates with the different affected YARS1 domains.
- Impaired protein translation is likely not the exclusive mechanism of ARS1-associated disorders.

**Supplementary Information:**

The online version contains supplementary material available at 10.1007/s00109-021-02124-9.

## Introduction

Pathogenic variants in aminoacyl-tRNA synthetases (ARS1) have been implicated in neurodevelopmental disorders and multisystem diseases affecting many different tissues. Aminoacyl-tRNA synthetases catalyze the attachment of specific amino acids to their corresponding tRNA. ARS1 proteins are cytoplasmic proteins, whereas ARS2 proteins act in the mitochondria.

*YARS1* encodes the cytosolic tyrosyl-tRNA synthetase (TyrRS), that is, responsible of linking tyrosine to its specific tRNA and requires homodimerization for enzyme activity.

Already in 1999, it was shown that apart from its enzymatic function, TyrRS has acquired at least two additional functional motifs in higher eukaryotes during evolution [1]. The N-terminal ELR motif was identified to play an important role as an interleukin-8 (IL-8)-like cytokine by binding to CXCR1, and to induce angiogenesis and thrombopoiesis [2, 3]. The C-terminal endothelial monocyte-activating polypeptide II (EMAP-II)-like domain exerts cytokine functions, e.g., by inducing the migration of leukocytes (macrophages, granulocytes) and the expression and release of tumor necrosis factor-$\alpha$ (TNF-$\alpha$), tissue factor or myeloperoxidase [1, 4, 5]. While the cytokine-like functions of TyrRS are inactive in the dimeric full-length protein, secretion, dissociation into monomers and cleavage by proteases (elastase or plasmin) activates the secondary functions of the N- and C-terminal fragments [6].

The spectrum of diseases associated with ARS1 is very broad. Pathogenic variants in all ARS1 coding genes have been implicated in autosomal recessive disorders, many of them presenting as early onset, severe multisystem diseases, while six of these (*GARS1*, *AARS1*, *KARS1*, *HARS1*, *MARS1*, *YARS1*) have also been implicated in autosomal-dominant, late-onset neuropathies [7–13].

Heterozygous pathogenic variants in *YARS1* have been first identified to cause autosomal-dominant Charcot-Marie-Tooth (CMT) neuropathy type C (OMIM #608,323) [7, 10]. Recently, five different biallelic variants in *YARS* have been published to cause autosomal recessive disorders [14–18]. However, the disorders attributed to biallelic pathogenic variants in *YARS* in these five families are heterogeneous ranging from severe intellectual disability (ID) with infant mortality to milder conditions without ID primarily affecting the sensory system [17].

The goal of this study was to delineate the clinical phenotypes associated with biallelic pathogenic variants in *YARS1* in order to improve disease recognition and health surveillance.

Via GeneMatcher and international collaborations, we identified and characterized a set of twelve patients with developmental delay and multisystem diseases that were all homozygous for the specific variant NM_003680.3:c.1099C > T, p.(Arg367Trp). We review all individuals with biallelic *YARS1* pathogenic variants reported in the literature and search for the common denominator and major discrepancies in clinical presentation of distinct *YARS1* variations.

By *in silico* analysis, we study the predicted effect of p.(Arg367Trp) on the protein structure and stability and discuss the impact on the canonical enzyme function of TyrRS and on the non-canonical, secondary functions of TyrRS.

We suggest that an impaired protein synthesis is not the primary mechanism underlying *YARS1*-(and *ARS1*-) associated disorders, but that they arise from defective non-canonical, secondary functional domains.

## Materials and methods

### Patient recruitment and clinical assessment

The study was performed according to the Declaration of Helsinki. The study was approved by the institutional ethical review boards (King Faisal Specialist Hospital and Research Center; Ref. No # 2,121,053 and 2,080,006; University Hospital Erlangen Ref. No. 253_15B). Patients with multisystem diseases and biallelic pathogenic variants in *YARS1* were recruited for the study. Ten of the 12 individuals with homozygous p.(Arg367Trp) *YARS1* variants were identified newly in-hospital or by international partners. Written informed consent for publishing of clinical, genetic data, and photographs was obtained from the patients and their legal guardians. Two (J:II-1 and K:II-2) of the 12 individuals had been identified within a large cohort of consanguineous families with ID before and clinical data that had not been published were provided after additional follow-up investigations and in depth review of medical records [15]. Clinical and laboratory findings of all patients were centrally reviewed, categorized and summarized.

### Identification of biallelic YARS1 pathogenic variants by exome sequencing and Sanger sequencing

Genetic testing preceding exome sequencing including cytogenetic and chromosomal microarray analyses did not reveal any causative genetic aberrations. Exome sequencing was performed according to standard methods (supplementary material). Sanger sequencing was performed for confirmation of reported variants.

### Review of published individuals

A literature search was performed in PubMed (search terms *YARS,* tyrosyl-tRNA synthetase deficiency). All clinical details and laboratory results were retrieved from the manuscripts, categorized and summarized.

### *In silico* variant prediction, protein modeling, and structural prediction

Effects of the p.(Arg367Trp) variant were predicted using PROVEAN, SIFT, PolyPhen-2, and MutationTaster [19]. Structural analysis of the p.(Arg367Trp) variant was based on a crystal structure of the C-terminal domain of human TyrRS (PDB code:1NTG) [20]. The effect of the pathogenic variant was modeled using Missense3D [21]. RasMol was used for visualization [22]. Multiple species protein alignment was done with Uniprot [23]. The impact of published recessive variants on protein structure and stability was analyzed using the VIPUR algorithm [24]. A VIPUR score > 0.5 indicates a critical destabilization of protein structure.

## Results

### Identification and *in silico* analysis of YARS1 c.1099C > T, p.(Arg367Trp)

**Fig. 1:**
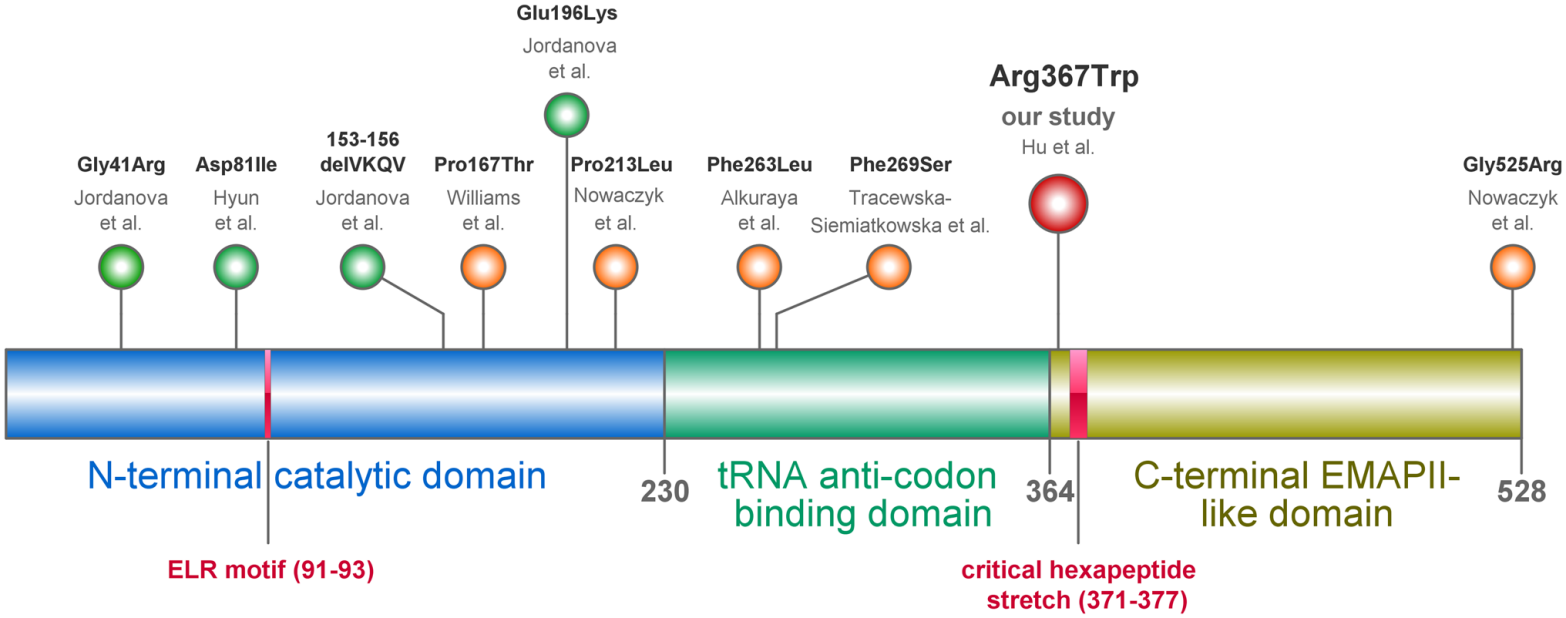
**a** Organization of the functional domains of the three domains of human TyrRS and biallelic variants reported in the literature. TyrRS has three domains: (i) The catalytic N-terminal domain is essential for aminoacylation of tRNA. As a monomeric fragment, the N-terminal domain has cytokine activity and the ELR motif is critical for this activity. (ii) tRNA anti-codon-binding domain. (iii) The C-terminal EMAP-II-like domain that was shown to be dispensable for aminoacylation. The heptapeptide sequence within this domain is critical for the cytokine activity. The homozygous variants p.(Pro167Thr) and p.(Pro213Leu) reside in the catalytic domain, harboring to the critical ELR motif, while most of the other variants reside outside this domain. All five heterozygous mutations causing Charcot-Marie-Tooth neuropathy reside in the catalytic domain. **b** Structure of the C-terminal domain of TyrRS shown as backbone representation indicating the elements of secondary structure. Arg367 is shown in space-filled presentation and colored according to the atom types. The heptapeptide sequence stretch critical for the cytokine activity (residues 371–377) is shown in blue. **c** Structural role of Arg367 in TyrRS: In the wild-type, Arg367 forms a salt-bridge to Asp478 (indicated by green dotted lines). **d** In the p.(Arg367Trp) mutant, the bulky uncharged tryptophan cannot form an electrostatic interaction resulting in domain destabilization. **c + d** Arg367/Asp478 are shown in stick presentation. **e** Multi-species amino acid alignment indicates that Arg367 is highly conserved in mammals and down to zebrafish (*Danio rerio*), but not in the western clawed frog (*Xenopus tropicalis*) [23]. **f** Amino acid alignment of the C-terminal EMAP-II-like domain of TyrRS compared to AIMP1. The corresponding residues Arg367 and Asp478 are conserved in both domains. TyrRS, tyrosine tRNA synthetase; AIMP1, aminoacyl-tRNA synthetase complex interacting multifunctional protein 1

In all included patients, homozygosity of the variant NM_003680.3(*YARS1*):c.1099C > T, p.(Arg367Trp) ([GRCH38/hg38] chr1:g.32,781,089G > A) in exon 10 of *YARS1* was identified by exome sequencing. No other likely pathogenic variant of clinical significance was identified in any patient. The haplotypes of the individuals originating from Saudi Arabia (C-I) differs from the haplotypes of individuals A + B (from Turkey) and L (from Puerto Rico) (Supplementary Table S1). While this finding does not rule out a founder effect common to the Saudi patients (C-I), it is less likely that also p.(Arg367Trp) of A + B and L traces back to the same, shared founder. The homozygous variant was absent from internal control databases but reported in dbSNP (rs376054085). The allele frequency in the gnomAD v2.1.1 population database is 3.89 × 10^–5^, with 11 known heterozygotes among 141,403 individuals, of which 10 have Latino and 1 has South Asian ancestry. The variant affects an arginine residue at position 367 (Arg367) of human TyrRS, in the C-terminal EMAP-II-like domain (residues 364–528) and is located three amino acids away from the tRNA anti-codon- binding domain (Fig. 1A). *In silico* tools predict deleterious effects on protein structure and function (prediction scores: PROVEAN: − 5.642, SIFT: 0.03, PolyPhen-2: HumDiv 1.000/HumVar 0.953, MutationTaster: “disease causing”). Arginine in position 367 is highly conserved in mammals and down to *Drosophila melanogaster*, but not in *Xenopus tropicalis* (western clawed frog) and *Caenorhabditis elegans* (Fig. 1E). Given the PS4, PP1, PP2, and PP3 ACMG/AMP criteria being fulfilled and the consistent phenotype in 12 individuals of 6 families, the p.(Arg367Trp) in *YARS1* was considered to be the causative genetic alteration in the patients [25].

### *In silico* protein modeling and structural prediction

The p.(Arg367Trp) variant is located in the C-terminal EMAP-II-like domain (residues 364–528) of TyrRS. *In silico* analysis of the wildtype structure reveals that arginine in position 367 (Arg367) forms tight electrostatic interactions with the oppositely charged aspartic acid in position 478 (Asp478), thereby stabilizing the domain structure (Fig. 1C). In the mutant, the bulkier and uncharged tryptophan (Trp367) cannot form this interaction resulting in a rearrangement of the sidechains and a reduced domain stability (Fig. 1D). The site of the p.(Arg367Trp) exchange is in the immediate vicinity of a hexapeptide stretch (residues 371–377) that is critical for the cytokine activity of the EMAP-II domain of TyrRS. [1] Amino acid alignment of the C-terminal EMAP-II-like domain of *YARS1* and the aminoacyl-tRNA synthetase complex interacting multifunctional protein 1 (AIMP1) shows that their corresponding residues Arg367 and Asp478 are conserved in both domains (Fig. 1F).

### Homozygous YARS1 c.1099C > T, p.(Arg367Trp) variant causes a distinct multisystem disease

We characterized the clinical features of the ten newly identified patients (families 1–4, 6) and two previously published patients (family 5) with homozygous p.(Arg367Trp) variants, all presenting with moderate ID and multisystem disease (Table 1, Fig. 1A–G) [15]. Family 1 originates from Turkey and family 5 originates from Iran close to the border to Turkey. Families 2, 3, and 4 stem from three different regions in Saudi Arabia. Family 6 originates from Puerto Rico.

**Table 1:** Clinical features of 12 individuals homozygous for NM_003680.3(*YARS1)*:c.1099C > T, p.(Arg367Trp). Features with a frequency reported $\ge$ 2 times were reported in this table

	**NM_003680.3(*YARS1)*:c.1099C > T, p.(Arg367Trp)**													
		**Family 1**		**Family 2**			**Family 3**			**Family 4**	**Family 5**		**Family 6**	
		**A:II-1**	**B:II-2**	**C:II-2**	**D:II-3**	**E:II-5**	**F:II-3**	**G:II-4**	**H:II-5**	**I:II-1**	**J:II-1**	**K:II-2**	**L:II-1**	
Sex		M	M	F	F	F	F	M	M	M	M	M	M	
Ethnicity		Turkish		Arabian			Arabian			Arabian	Iranian		Puerto-Rican	
Age at last exam (years)		15	9	5	14	11	7	6	3	4	15	13	4	
	**Body measurements**													
Microcephaly		+	+	+	+	+	+	+	+	+	+	+	+	12/12 (100%)
Birth (weeks)		39	40	39	n/r	n/r	40	40	40	39	38	38	40	
- weight at birth (g)/(SD)		2600/ − 2.0	2910/ − 1.7	3000/-0.8	n/r	n/r	2/ − 2.8	n/r	n/r	2700/ − 1.8	n/r	n/r	3285/-0.77	
-Length at birth (cm)/(SD)		49/-1.26	48/ − 2.0	n/r	n/r	n/r	n/r	n/r	n/r	n/r	50/ − 0.06	50/ − 0.06	49.5/-1.3	
-OFC at birth (cm)/(SD)		34/ − 1.0	33/-2.0	n/r	n/r	n/r	n/r	n/r	n/r	n/r	35/ − 0.4	35/ − 0.4	33/-2.0	
Failure to thrive **Age of measurement (y)**		+ 6 3/12	+ 7 6/12	+ 5 7/12	+	n/r	+ 3/12	+	+	+ 4 5/12	+ 14	+ 13	+ 3 6/12	11/12 (92%)
-Weight (kg)/(SD)		15/ − 2.9	17.3/-3.1	12.6/ − 2.7	n/r	n/r	1.7/ − 5.4	< 3^rd^	< 3^rd^	12.2/ − 3.2	n/r	n/r	12.3/ − 1.8	
-Length (cm)/(SD)		108/ − 3.3	112/ − 2.7	102/ − 3.6	n/r	n/r	< 3^rd^	< 3^rd^	< 3^rd^	94/ − 3.0	122/ − 5.1	120/ − 4.6	94/ − 1.4	
-OFC (cm)/(SD)		48/ − 3.6	47/ − 4.4	45/ − 5.5	n/r	n/r	< 3^rd^	< 3^rd^	< 3^rd^	45/ − 5.1	44/ − 6.7	47/ − 4.8	47/ − 3.1	
	**Cognitive development and acquired skills**													
Intellectual disability (IQ)		+	+	+	+	+	+	+	+	+	+ (30)	+ (30)	+	12/12 (100%)
Developmental delay		+	+	+	+	+	+	+	+	+	+	+	+	12/12 (100%)
Ability to sit alone—age		2 y	n/r	n/r	n/r	n/r	2.5 y	2 y	2	1 y	1 y 8 m	1 y 8 m	1 y	8/8 (100%)
Ability to walk—age		3 y	3.5 y	3 y	3 y	2 y	No (7)	4 y	No (3)	No (4)	4 y	4 y	2.5 y	9/12 (72%)
Speech—single words		3.5 y	3 y	4 y	4 y	3 y	6 y	5 y	2–3 y	2 y	2 y	2 y	No	11/12 (92%)
Speech—simple sentences		8.5 y	No	No	No	No	No	No	No	No	No	No	No	1/12 (8%)
Follows simple demands		+	+	+	+	+	No	No	No	No	No	No	No	5/12 (42%)
	**Neurological findings**													
Muscular hypotonia		+	+	-	-	-	+	+	+	+	+	+	+	9/12 (75%)
Ataxia		+	+	+	+	+	-	-	-	n/r	+	+	n/r	7/10 (70%)
Poor coordination		+	+	n/r	n/r	n/r	-	-	-	n/r	+	+	+	5/8 (63%)
Facial hypotonia		+	+	-	-	-	+	n/r	n/r	-	+	+	+	6/10 (60%)
Seizures		-	-	-	-	-	-	-	-	-	-	-	-	0/12 (0%)
	**Liver**													
Hepatomegaly		n/r	+	n/r	n/r	n/r	+	+	+	+ (mild)	+	+	n/r	7/7 (100%)
Hyperechogenic liver texture		n/r	+	n/r	n/r	n/r	+	+	+	+ (mild)	-	-	n/r	5/7 (71%)
Elevated liver enzymes		-	-	-	n/r	n/r	-	-	-	One episode	n/r	n/r	-	1/8 (13%)
History of Ascites		-	-	-	-	-	-	-	-	-	+	-	-	1/12 (9%)
Liver failure		-	-	-	-	-	-	-	-	-	-	-	-	0/12 (0%)
	**Abnormal laboratory findings**													
Chronic anemia		+	+	+	n/r	n/r	+	+	+	+	n/r	n/r	+	8/8 (100%)
Hypothyreoidism		+	-	-	-	-	+	+	+	-	n/r	n/r	n/r	4/9 (44%)
Growth hormone deficiency		+	-	n/r	n/r	n/r	n/r	n/r	n/r	n/r	n/r	n/r	n/r	1/2 (50%)
	**Brain imaging (MRI)**													
Age		12 2/12	1 6/12	1.5 y	2 y	n/r	1 y/3 y	2 y	2 y	2 y	n/r	n/r	n/r	9/11 (81%)
Thin corpus callosum		+	+	-	-	-	+	+	+	+	n/r	n/r	n/r	6/9 (67%)
Reduced brain volume		+	+	-	+	-	+	+	+	+	n/r	n/r	n/r	7/9 (78%)
Delay in myelination		+	+	-	-	-	+	+	+	+	n/r	n/r	n/r	6/9 (67%)
Other findings		-	+ (#)	-	-	-	-	+ ($)	-	-	n/r	n/r	-	2/9 (11%)
	**Nerve conduction—sensory potentials**													
Decreased velocity		-	-	n/r	n/r	n/r	n/r	n/r	n/r	n/r		-	n/r	0/4 (0%)
Decreased amplitude		-	-	n/r	n/r	n/r	n/r	n/r	n/r	n/r		-	n/r	0/4 (0%)
	**Other findings**													
Visual impairment		-	-	-	-	-	-	-	-	-	n/r	n/r	-	0/11 (0%)
Retinal degeneration		-	-	-	n/r	n/r	n/r	-	-	-	n/r	n/r	-	0/6 (0%)
Hearing loss		-	-	-	-	-	+	-	-	n/r	-	+	-	2/12 (17%)
Vomiting		-	-	-	-	-	+	-	-	-	-	-	+	2/11 (18%)
Gastroesophageal reflux		-	-	-	-	-	+	-	-	-	-	-	+	2/11 (18%)
Dyspnea		-	-	-	-	-	+	n/r	n/r	-	-	-	-	1/10 (10%)
Chest X-ray		n/r	n/r	Normal			+ (##)	n/r	n/r	+ (ß)	n/r	n/r	n/r	
Recurrent bronchitides		+	-	-	-	-	-	-	-	-	-	-	-	1/12 (8%)
pancreatitis		-	-	-	-	-	-	-	-	-	-	-	+	1/12 (8%)
Cardiovascular/urinary abnormalities		-	+ (&)	-	-	-	+ ( +)	-	-	-	-	-	-	2/12 (17%)
Recurrent infections		-	-	-	-	-	+ (§)	-	-	-	-	-	-	1/12 (8%)
**Mortality—age (years)**											15			

^#^Arachnoid cyst 6 × 3 × 3 cm

^$^periventricular leucomalazia

^&^hydro-nephrosis of right kidney

^+^PDA, muscular ventricular septal defect

^##^hypoplastic right lung

ß, perihilar bronchial wall thickening

*MRI*, magnet resonance imaging

*OFC*, occipitofrontal circumference

*n/r*, not reported

SD, standard deviation

The individuals were born at term. Body measurements at birth, including weight, length, and head circumference, were at the lower end of the range. The weight at birth ranged between − 0.8 and − 2.8 standard deviations (SD) (5/5) (medium − 1.8 SD).

**Fig. 2:**
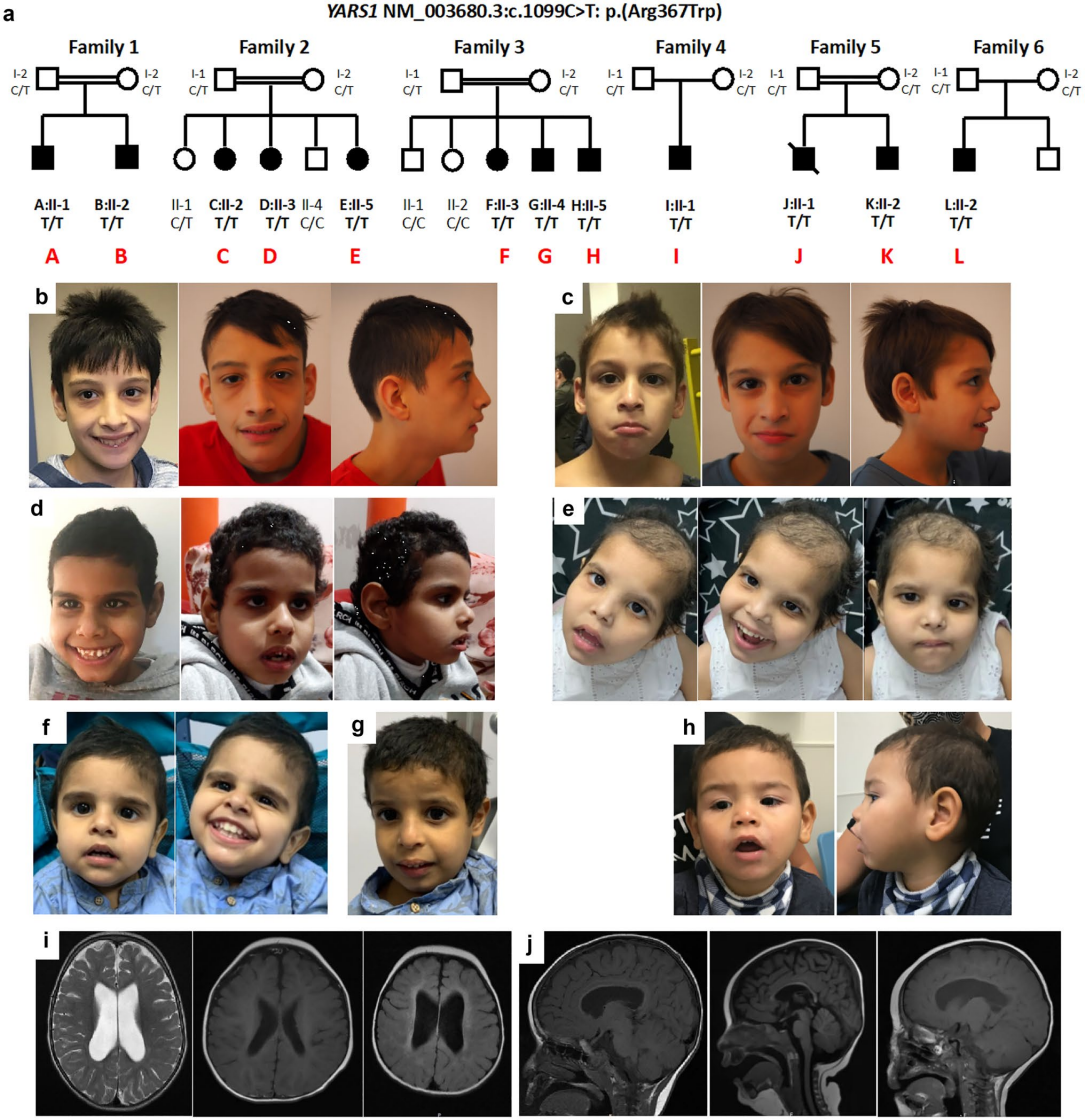
**a** Pedigrees of affected individuals with homozygous p.(Arg367Trp) in *YARS1.* Individuals J and K had been reported within a large cohort of consanguineous families with ID [15]. Individual L additionally has two unaffected half-siblings (not depicted). **b–h** Facial features of individuals (A + B, F + G + H, I, and L). **b** Individual A (family 1:II-1) at age of A 11 and 15 years, **c** Individual B (family 1:II-2) at age of 7 and 9 years, **d** Individual I (family 4:II-1) at age of 7 years, **e** Individual F (Family 3:II-3) at age of 3 years, **f** Individual H (family 3:II-5), **g** Individual G (family 3:II-4) at age of 5 years. **h** Individual L (Family 6:II-41) at age of 3.5 years. Facial dysmorphic features including deep set eyes (**b, c, d, g, h**), sparse hair (**e–h**), a long nose (**b-d**), a flat nasal bridge (**b–d**), full cheeks (E − G), long columella (**c–d, f–g**), flat philtrum (**b–h**), open mouth appearance (**b, d–h**), low set (1), and large ears (**b–d**, **h**), together resembling progeroid-like appearance (**b–h**). **i** Axial sequences (head MRI) of individuals I, G, and F showing wide lateral ventricles as a sign of diffuse cerebral volume due to periventricular white matter loss, **j** Sagittal sequences (head MRI) of individuals I (2y), G (2y), and F (2y) (from left to right) showing thinning of corpus callosum

All individuals, of whom data were available, had acquired microcephaly (12/12, 100%), a postnatal failure to thrive or growth delay (11/12, 92%) and developmental delay or intellectual disability (12/12, 100%). Most individuals learned to walk independently (9/12, 75%, average age 3.3 years, SD 0.7) and all acquired the ability to communicate using single words (11/12, 92%, average age 3.4 years, SD 1.3). At the age of last evaluation, almost half of the individuals were able to follow simple demands (5/12, 42%), but only one individual spoke simple sentences of three or more words (1/12, 8%, 8.5 years). Neurological assessment revealed muscular hypotonia (9/12, 75%) including the face (6/10, 60%), ataxia (7/10, 70%), and a poor coordination (5/8, 63%). None of the patients had a history of epilepsy. Seven patients were reported to have abnormal liver findings including hepatomegaly (7/7, 100%), hyperechogenic liver texture on ultrasonography (5/7, 71%), or an episode of elevated liver enzymes (1/11, 9%) suggestive of stable liver disease with steatosis. Serum albumin was in the lower normal reference range (mean $\pm$ SD, 3.4 $\pm$ 0.5 g/L). Until the last investigation (oldest age 15 years), only one patient had a history of temporary ascites and no patient showed clinical signs of liver failure. All individuals who had a blood analysis done displayed a chronic microcytic anemia (8/8, 100%, mean $\pm$ SD, hemoglobin 10.9 $\pm$ 0.9 g/dl, MCV 63.9 $\pm$ 2.2 fL, MCH 19.6 $\pm$ 1.4 pg). Almost half of patients had laboratory results suggestive of hypothyroidism (4/9, 44%). Sporadic findings and features included mild hearing impairment (1/12, 8%), gastroesophageal reflux and vomiting (2/12, 17%), abnormal findings on chest X-ray (2/11), dyspnea (1/12, 8%), recurrent obstructive pulmonary disease (1/12, 8%), and recurrent infections (1/12, 8%). MRI imaging of the head revealed a reduced brain volume, (7/9, 78%), a thin corpus callosum (6/9, 67%), and a delay in myelination (6/9, 67%) (Fig. 2 H + J). Patients displayed facial dysmorphic features including a flat philtrum (6/7, Fig. 2 B–G), a open mouth appearance (6/7, Fig. 2 B, D, E, F, G), deep set eyes (5/7, Fig. 2 B, C, D, G), hanging columella (5/7, Fig. 2B, C, D, F, G), a prominent nose tip with relative small nares (3/7, Fig. B, C, D), low set (1/7, Fig. 2F), and large ears (4/7 Fig. B, C, D), sparse hair (4/7, Fig. 2 E–G), together reminding of a progeroid-like appearance (6/6, Fig. 2 B–G).

In summary, the homozygous missense variant p.(Arg367Trp) causes a clinically consistent, multisystem disease with mildly delayed motor and severely impaired speech development, microcephaly, failure to thrive, short statue, muscular hypotonia, ataxia, brain atrophy, microcytic anemia, hepatomegaly, hypothyroidism, and facial features including a deep set eyes, a flat philtrum, and open mouth appearance. Clinical heterogeneity is only observed for hypothyroidism (approximately 50% of patients affected) and some sporadic findings such as hearing loss or gastroesophageal reflux.

*YARS1* is one of the established disease genes of CMT. Patients and their parents had no peripheral palsy, impairment of peripheral sensation, autonomic dysfunction, or neuropathic pain. In conclusion, no clinical signs of peripheral neuropathy were present. In four patients, peripheral nerve conduction studies were performed which showed unremarkable amplitudes of sensory action potentials and normal nerve conduction velocities. Detailed case reports medical histories are reported in the supplementary material.

### Review of individuals from the literature: allelic and clinical heterogeneity associated with biallelic variations in YARS1

In the recent literature, five homozygous or compound heterozygous disease-causing variants in *YARS1* other than p.(Arg367Trp) have been described (Table 2).

**Table 2:** Summary of all patients with biallelic variants in *YARS1* other than p.(Arg367Trp) from the literature. *In silico* analysis with VIPUR algorithm predicts that all biallelic variants associated with recessive multisystemic disease significantly impact the protein structure and stability (score > 0.5) of the respective TyrRS protein domain

	Nowaczyk et al. 2016		Tracewska-Siemiatkowska et al., 2017	Williams et al., 2019		Alkuraya et al., 2019	**This study**	
	2 patients		1 patient	7 patients		1 patient	12 patients	
Gender	1F/1 M		F	5F/2 M		F	4F/8 M	
Origin	Polish		Swedish	Amish (USA)		Saudi Arabian	Turkish/Iranian/Saudia Arabian/Puerto-Rican	
Variant	p.(Pro213Leu) p.(Gly525Arg) *		**p.(Phe269Ser)**	**p.Pro167Thr**		**p.(Phe263Leu)**	**p.(Arg367Trp)**	
Domain	N-terminal catalytic domain and C-terminal EMAP-II-like domain		tRNA anti-codon-binding domain	N-terminal catalytic domain		tRNA anti-codon-binding domain	C-terminal EMAP-II-like domain	
VIPUR Score	0.84 0.93		0.86	0.73		0.67	0.90	
Pregnancy/birth	Normal No complications		Normal No complications	3/7: premature 5/7: placental abnormalities 4/7: growth restriction		Primordial dwarfism	Body measurements at birth in lower range	
Short statue	+	1/2	+	+	7/7	+	+	9/9
Failure to thrive	+	2/2	+	+	7/7	+	+	10/12
Microcephaly	+	1/2	-	+	7/7	+	+	12/12
Developmental delay	±	1/2 ^A^	-	+	7/7	+	+	12/12
Muscular hypotonia	+	2/2	-	+	7/7	n/r	+	9/12
Poor coordination	-	0/2	+	+	7/7	n/r	+	5/8
Hearing loss	-	0/2	+	+	7/7	n/r	±	2/11

^+^Present in > 50%; ^±^present in 5–50%; ^−^absent

^*^compound heterozygous

^A^Mild

^B^at age of 7 months after anoxic brain injury

^C^steatosis

^D^cirrhosis

^E^cystic changes

^F^dysmyelination, diffusion restriction, T2 hyperintensity

^G^during first 2 years; *n/r*, not reported

In 2016, two siblings with compound heterozygous likely pathogenic variants c.638C > T, p.(Pro213Leu) and c.1573G > A, p.(Gly525Arg) in *YARS1* were reported. They were affected by mild developmental delay (1/2) or normal development (1/2), failure to thrive in the first year of life, short statue, microcephaly (1/2), stable liver disease with steatosis (without inflammation), hypercholesterinemia, areflexic muscular hypotonia (1/2), cystic lung disease, and non-progressive mild brain atrophy and thinning of corpus callosum with cystic changes in periventricular white matter [18]. The nerve conduction was not affected and both parents had no evidence of neuropathy or neurological disease.

In 2017, the homozygous variant c.806 T > C, p.(Phe269Ser) in *YARS1* in one individual was associated with poor weight gain (necessitating tube feeding), severe visual impairment caused by progressive-rod-cone degeneration (fundus pigmentation), profound hearing impairment, poor balance and muscular hypotonia during first years of life, stable liver disease with steatosis (later minor fibrotic changes), primary amenorrhea, but normal psychomotor development [14]. MRI images revealed a thin corpus callosum. Laboratory analyses showed high level of blood platelets related to hyperactive bone marrow.

In 2019, seven related individuals with a homozygous c.499C > A, p.(Pro167Thr) pathogenic variant within the catalytic N-terminal domain of *YARS1* were reported to be affected by a more severe multisystem disorder than previously reported [17]. All patients had developmental delay, microcephaly, bilateral sensorineural hearing loss, and poor growth. Some patients had abnormal ophthalmological findings including pigmentary degeneration and visual impairment. They were affected by exocrine pancreatic insufficiency and chronic, progressive liver disease with steatosis at the early stages, and inflammatory cirrhosis at the later stages. MRI images of the head revealed restricted diffusion among the white matter and abnormal T2-weighted hyperintensity with dysmyelination. Autopsy of one of the deceased patients showed evidence of chronic neuronal loss with vacuoles. Laboratory analyses showed chronic anemia, hypalbuminemia, and intermittent proteinuria. Four patients died during the first 2 years of life due to progressive liver failure.

In 2019, in a large cohort of patients with microcephaly, the homozygous variant c.789C > A,p.(Phe263Leu) in *YARS1* was associated with microcephaly, developmental delay and primordial dwarfism in one girl [16].

In summary, recessively inherited variants in *YARS1* cause some overlapping clinical features including small stature and motor problems (all published variants). There are also decisive differences between clinical features associated with the different variants.

The neurological impairment of p.Pro167Thr, p.(Arg367Trp), and p.(Phe263Leu) is much more severe when compared to p.(Phe269Ser) or the compound heterozygous variants p.(Pro213Leu) and p.(Gly525Arg). For almost all variants, some liver involvement has been reported, ranging from mild steatosis (p.(Pro213Leu)/p.(Gly525Arg); p.(Phe269Ser); p.(Arg367Trp)) to cirrhosis and hepatic failure (p.Pro167Thr). Abnormal blood cells (anemia or low platelets) are reported in association with p.(Phe269Ser), p.Pro167Thr, and p.(Arg367Trp). Hearing and visual impairment is only consistently observed for p.(Phe269Ser) and p.Pro167Thr. The disease severity associated with p.(Arg367Trp) (the variant of this study) lies somewhere in between with p.Pro167Thr and the severe end and p.(Pro213Leu), p.(Gly525Arg), and p.(Phe269Ser) at the milder end of the spectrum. The variants reside in different protein domains. *In silico* analysis with VIPUR algorithm predicts that all biallelic variants associated with recessive multisystem disease significantly affect the protein structure and stability (score > 0.5, Table 2), which leads to a significant disruption of the specific protein domain in which they reside [24]. Given that the variants are located in different protein domains the functional impact of the structural domain destabilization is expected to differ between the variants (Fig. 1). The variants p.(Phe269Ser), p.(Pro213Leu), p.(Phe263Leu), and p.(Gly525Arg) were absent from gnomAD and the p.(Pro167Thr) was detected only twice (in European) within at least 141,000 individuals. Compared to the biallelic variants reported in the literature, p.(Arg367Trp) was observed in the heterozygous state in eleven individuals from gnomAD v2.1.1 (1 South Asian and 10 Latino) [26].

## Discussion

Since the availability and broad use of exome sequencing, the success rate in solving the genetic cause of ID and multisystem diseases has significantly increased. Most of the new disease genes have been identified as private disease-causing variants in single families within larger cohorts [15, 16, 27]. For many disease genes, reliable clinical data on the features and course of the associated disorders are sparse. For appropriate patient counseling and guidance, the delineation of typical clinical signs is needed.

Aminoacyl-tRNA synthetases link specific amino acids with their transfer RNAs and play a key role in protein translation. Beyond their canonical role, many ARS1 acquired secondary functions by the incorporation of additional domains during evolution of more complex eukaryotes and play key regulatory roles e.g. in immunomodulation and inflammation [28–31].

TyrRS couples the amino acid tyrosine to its specific tRNA. In this study, we identified and characterized 10 newly diagnosed and two previously reported individuals carrying the homozygous missense variant NM_003680.3:c.1099C > T, p.(Arg367Trp) in *YARS1* and summarized all previously published patients from the literature, carrying alternative, biallelic variants in *YARS*. We identified a specific multisystem disease affecting not only the central nervous system, but also the liver, the hematopoietic system (anemia), and the endocrine system (hypothyroidism). The specification of the common, clinical features allows to establish the recommendation of monitoring affected individuals with regards to psychomotor, liver, hematological, and hormonal symptoms. In addition, the identification of total 12 patients with a matching and common clinical phenotype confirms the classification of the variant p.(Arg367Trp) to be unequivocally pathogenic.

We originally intended to include patients with multisystem disorders caused by biallelic variants in *YARS1*, regardless of the specific variants. Interestingly, our efforts that included GeneMatcher and international collaborations, only identified additional patients that were homozygous for the p.(Arg367Trp) variant. We therefore compared the allele frequency of the published variants to the frequency of p.(Arg367Trp) in population databases. p.(Arg367Trp) and the other variants from the literature have not been identified in any of the control individuals from the Iranome or GME Variome projects [32, 33]. In gnomAD v2.1.1, the higher allele frequency of p.(Arg367Trp) (11 among a total of more than 141,000 individuals) compared to variants from the previous publication (0–2 alleles among more than 141,000 individuals) in the general population probably accounts for the higher prevalence of homozygous p.(Arg367Trp) and explains why we identified an unexpected and disproportionate high number of patients with this specific variant [26]. Despite predominant reports of heterozygotes in the Latino population, we only recruited one patient originating from Latin America (Patient L, Puerto Rico). A possible reason might be the relatively rare application exome sequencing in Latin America, or the predominant random-mating scheme in contrast to the prevalent consanguineous mating which is common in Middle East and South Asia. Of note, many variants causing autosomal recessive disorders in communities with high consanguinity rates occur as private disease-causing variants in single families. This also seems to be the case for the previously published individuals with variants that are absent from established control databases. In contrast, the variant p.(Arg367Trp) is reported with a much higher frequency and, therefore, might be of high and notable clinical relevance. Haplotype analysis of our patients revealed diverging haplotypes surrounding the *YARS1* gene region suggesting that p.(Arg367Trp) does not essentially trace back to a single founder.

As a native protein TyrRS comprises three functional domains: the catalytic domain, the anticodon-binding domain, and the C-terminal domain. TyrRS is only catalytically active as a homodimer [34–36]. While the full-length, dimeric TyrRS (528 AA) has no known additional cytokine activity, proteolytic cleavage and dissociating of monomeric TyrRS into the N-terminal TyrRS^Mini^ (composed of the catalytic and anticodon domain) and a C-terminal EMAP-II-like domain (164 AA) activates the secondary functions of these domains [1, 2].

Many genes involved in protein synthesis have been associated with neurodevelopmental disorders [37, 38]. In view of the core function of TyrRS in aminoacylation, it is tempting to expect that a limited protein synthesis which might not meet translational demand causes *YARS1*-associated disorders. Based on our findings, the hypothesis conferring that a dysbalanced protein homeostasis is the sufficient explanation of *YARS1*-associated disorders must be questioned for several reasons. First, the hypothesis of impaired protein synthesis lacks to explain the considerable clinical variability of autosomal recessive diseases associated with *YARS1* and also other ARS1-deficiencies. If a reduced or less specific enzyme activity was the common cause of disease, we would expect a more homogeneous clinical presentation of all biallelic *YARS1* and other ARS1 pathogenic variants. Second, pathogenic variations causing recessive ARS1 deficiencies generally reside in catalytic or anticodon binding domains of ARS1 genes. However, *in silico* analysis show that p.(Arg367Trp) lie outside these catalytically important domains, but that they are located in the C-terminal domain that accounts for non-canonical, immunomodulatory functions [18].

It was shown that the cleaved N-terminal TyrRS^Mini^ retains the aminoacylation activity of native TyrRS, and thus, the C-terminal EMAP-II-like domain was shown to be dispensable for aminoacylation [1, 39]. Therefore, p.(Arg367Trp) that resides in the C-terminal domain probably does not affect the aminoacylation activity of TyrRS. *In silico* analyses show that Arg367 is critical for a tight electrostatic interaction with the oppositely charged Asp478 and that p.(Arg367Trp) reduces the domain stability of the C-terminal EMAP-II-like domain which is in close vicinity of the critical hexapeptide stretch of this domain (residues 371–377). As a consequence, the destabilization of the protein structure could disrupt the EMAP-II-like cytokine activity. EMAP-II is also known as the aminoacyl-tRNA multiple synthetase complex (MSC) Interacting Multifunctional Protein 1 (AIMP1). The EMAP-II-like domain of *YARS1* shares 51% identity (78% similarity) including Arg367 (Fig. 1F). It plays a role in the assembly of the multiple synthetase-complex (MSC), and once it is secreted from apoptotic cells confers multiple effects on angiogenesis, wound healing, glucose metabolism, and neuronal development [40]. Of note, Arg367 and Asp478 are not only highly conserved in the C-terminal domain of TyrRS, but also its corresponding residues in the homologue EMAP-II underpinning their importance for the protein function (Fig. 1F). Homozygous pathogenic variants in *AIMP1* have been reported to cause moderate to severe intellectual disability suggesting that imbalances in EMAP-II-like functions are sufficient to cause developmental disorders [41, 42].

Apart from its procytokine activity, the C-terminal domain functions to sterically block the critical chemotactic ELR motif in the N-terminal domain by mutual shielding [43]. By this mechanism, the C-terminal domain is thought to suppress the cytokine activity of TyrRS in the state of a native full-length protein [43]. Thus, destabilization of the C-terminal domain could impede the steric block of the ELR motif and thus induce a proinflammatory phenotype. The finding of hyperechogenic liver cirrhosis in our patients and patients from the literature, potentially supports the idea that an imbalanced immune response and excessive inflammation may play a role in the underlying disease pathophysiology. The finding of chronic, microcytic anemia in relation to normal ferritin and low reticulocytes could also be explained by a chronic inflammatory condition.

The phenotypic spectrum of the disorder associated with p.(Arg367Trp) overlaps, but also differs to a certain extent from other reported pathogenic variants in *YARS1* from the literature. *In silico* analysis of the protein structure predict a significant impact of all previously reported recessive variants on the stability of the respective TyrRS protein domain.

Compared to the homozygous variant p.(Pro167Thr) that causes a severe multisystem disorder including the liver, the pancreas and the kidneys and a high infant mortality, p.(Arg367Trp) is associated with a milder clinical presentation [17]. In contrast, the compound heterozygous variants p.(Pro213Leu)/p.(Gly525Arg) and p.(Phe269Ser) were described to compromise the auditory perception, eyes, reproductive organs, and liver, while not causing ID [14]. These observations suggest that *YARS1* displays considerable allelic heterogeneity concerning the disease severity and pattern. p.(Pro167Thr) at the severe end of the spectrum and p.(Pro213Leu) at the mild end of the spectrum both reside in the N-terminal catalytic domain of *YARS1*, while p.(Arg367Trp) with a disease severity in between these both domains is located in the C-terminal domain. Given the limited number of variants described so far and the phenotypic heterogeneity associated with the different domains, it is difficult to draw a clear link between the affected domain and the disease severity and organs involved.

The hypothesis that autosomal recessive disorders associated with *YARS1* are caused by dysregulated secondary functions rather than only by impaired protein synthesis is further supported by location of the variations reported in patients from the literature outside the TyrRS catalytic domain (e.g., p.(Gly525Arg) in the C-terminal domain).

Until recently, all 19 ARS1 have been associated with autosomal recessive diseases [8]. While recessively inherited ARS1 disorders share some common clinical signs, e.g., involvement of the CNS and microcephaly (in all but *HARS1*), there is a striking heterogeneity of the clinical manifestation which does not follow a reproducible pattern. Liver involvement has been reported for *IARS*, *LARS*, and *MARS* while for example skin anomalies have only been described for *QARS* or anatomical heart anomalies have only been described for *MARS* [38, 44–49]. *LARS1*- and *MARS1*-associated disorders are associated with neurological impairment, MRI abnormalities, liver disease, anemia, and endocrine abnormalities and show the greatest clinical overlap with the *YARS1* p.(Arg367Trp)-associated phenotype delineated here.

Another example of ARS1-associated disorders, in which an impaired protein synthesis as the causative disease mechanism can be questioned is *VARS1* causing developmental delay with microcephaly [38]. Interestingly, in in vitro assays, the authors found a 50% residual aminoacylation activity. Because reductions in enzyme activity of approximately 50% are often well tolerated, it can be speculated that reduced aminoacylation is not the underlying disease mechanism, but that dysregulated secondary functions (for example, dysregulation of VEGF) might be involved. In many ARS1 genes, over 200 “catalytic nulls” natural splice variants have been annotated which primarily ablate or disrupt the catalytic domain but retain the noncatalytic section. This observation underpins the diverse, functions of nonenzymatic domains of ARS1 genes [50].

CMT is another disease reflecting the significance of secondary protein functions of *YARS1*. Since the discovery of pathogenic variants in *YARS1* causing CMT type C more than 15 years ago, the exact disease mechanism has not been understood, yet. All five CMT-causing mutations in *YARS1* reside in the N-terminal catalytic domain (Fig. 1A). Because aminoacylation activity is not a shared property of pathogenic variations, it is unlikely that haploinsufficiency affecting the aminoacylation enzyme activity is the underlying mechanism [7, 51]. Currently, gain-of-function pathogenic variants in non-catalytical domains or transcriptional dysregulation are discussed to be the potential underlying disease mechanism [52]. Of note, none of the patients or parents reported here is affected by CMT neuropathy. This is in line with the absence of neuropathy in patients with recessive disorders caused by other ARS1 genes which have been implicated with CMT [8].

One limitation in the interpretation of the impact of *YARS1* variants on protein function is that to date no structural model of the full-length protein is available and that separate structures of mini-TyrRS and C-domains are the basis for functional predictions. Another limitation is that our assumptions are based on clinical findings, *in silico* analyses, and on recent findings from functional in vitro studies. Protein dimerization assays and yeast growth complementation assays have been performed for *YARS*1 p.(Pro167Thr) showing impaired dimerization and limited yeast growth [17]. Functional studies for p.(Arg367Trp) have not been performed. As the C-terminal domain was shown to be dispensable for aminoacylation, it can be supposed that p.(Arg367Trp) residing in the C-terminal domain does not affect the enzymatic function of TyrRS; however, indirect effects on enzymatic function cannot be ruled out [1, 39]. Experimental studies will be required to systematically investigate the impact of all variants on enzyme activity and to delineate the disease mechanism.

In conclusion, the characterization of the distinct multisystem disease associated with p.(Arg367Trp) and other *YARS1* biallelic variants will help in the clinical diagnostic-workup of undiagnosed patients and will improve counseling of affected families. There is decisive clinical heterogeneity associated with different variants in *YARS1* and also across different ARS1-disorders. An advanced understanding of secondary functions of ARS1 will pave the way to identify new targets for treatment of ARS1-associated multisystem disorders and CMT in the future.

## Supplementary Information

Below is the link to the electronic supplementary material.

Supplementary file1 (XLSX 11 KB)

## Data Availability

Additional clinical data and materials will be individually available upon request.
